# Influence of antibiotic resistance on disinfectant tolerance of Escherichia coli, Staphylococcus aureus, Enterococcus faecium and Campylobacter jejuni

**DOI:** 10.1099/acmi.0.001098.v4

**Published:** 2025-12-22

**Authors:** Emma Davies, Marie Lindridge, Rebecca J. Gosling, Richard Piers Smith, Claire Oastler

**Affiliations:** 1 Department of Bacteriology, Animal and Plant Health Agency (APHA – Weybridge), Addlestone, KT15 3NB, UK; 2 Health and Safety Executive (HSE), Harpur Hill, Buxton, Derbyshire SK17 9JN, UK; 3 Department of Epidemiological Sciences, Animal and Plant Health Agency (APHA – Weybridge), Addlestone, KT15 3NB, UK

**Keywords:** antibiotic resistance, biocides, *Campylobacter jejuni*, *Enterococcus faecium*, *Escherichia coli*, *Staphylococcus aureus*

## Abstract

Antimicrobial-resistant (AMR) bacteria are an increasing concern for human and animal medicine. As a result, biosecurity measures such as cleaning and disinfection are becoming heavily relied upon to eradicate and control AMR pathogens. However, evidence of co- and cross-resistance between antimicrobials and disinfectants is rising. The influence of AMR on disinfectant tolerance is poorly understood for pathogens of veterinary and public health importance. Therefore, this study aimed to compare disinfectant tolerance of fluoroquinolone-resistant *Campylobacter jejuni*, livestock-associated methicillin-resistant *Staphylococcus aureus*, multi-drug-resistant *Escherichia coli* and vancomycin-resistant *Enterococcus faecium*, with their antibiotic-susceptible counterparts. *In vitro* disinfectant efficacy was assessed, in the presence of organic matter, against a panel of eight disinfectants from six classes. The disinfectant efficacy varied widely depending on bacterial species and disinfectant class. Furthermore, approved disinfectant concentrations were not always deemed effective. All four bacterial species were typically most susceptible to aldehyde and/or quaternary ammonium compound-based products. Mixed evidence was found to suggest a role of AMR in disinfectant tolerance; no role of AMR was identified in *E. coli*, *C. jejuni* or *E. faecium*, whereas a potential role was identified in *S. aureus*.

## Data Summary

The authors confirm that all supporting data, code and protocols have been provided within the article.

## Introduction

The use of antimicrobial drugs in human and animal healthcare is widespread. Often, their application has been inappropriately directed, such as for growth promotion in farming practices [1]. As a consequence, a selection pressure is created, favouring the survival of resistant bacteria [1]. The interconnection between humans and animals through the food chain, direct contact and environmental contamination exacerbates the threat posed by antimicrobial resistance pathogens [1,2]. Resistance, particularly to antibiotics, is a major concern for human, animal and environmental health. The World Health Organization (WHO) produced a list of high-priority critically important antibiotics (HP-CIA), which includes aminoglycosides, third- and fourth-generation cephalosporins, fluoroquinolones, glycopeptides, macrolides and colistin [3]. Over recent decades, within the UK and European Union (EU), the use of antibiotics has been restricted, with legislative bans enforced on the use of antibiotics for routine use, growth promotion, prophylaxis and meta-phylaxis [4,5]. A consequence of this is an increased importance of biosecurity measures, such as restricted access to farm premises, waste management, various husbandry practices and cleaning and disinfection (C and D) procedures, which have been highlighted to help active prevention of persistence and dissemination of antimicrobial resistance (AMR) [6].

Disinfectants are widely used on livestock premises and in food production sectors for surface disinfection. They are a group of biocides (chemical substances used with the intention to inactivate harmful micro-organisms), specifically used with the aim to affect organisms present [7]. They may have bacteriostatic (inhibit further growth) or bactericidal (total kill) activity [8].

AMR continues to persist despite the reduction in usage of antimicrobial drugs on European farms and an increase in the application of biosecurity practices [9,10]. Evidence of co- and cross-resistance between antibiotics and other antimicrobials, such as disinfectants, gives rise to concerns over the efficacy of C and D measures [11,12]. Co-resistance may occur when AMR genes and disinfectant tolerance genes (such as those regulating efflux or biofilm formation) are located on the same mobile genetic element, such as a plasmid [11,12]. On the other hand, cross-resistance occurs when the same mechanism, for example, a gene involved in efflux, leads to altered susceptibility against both antibiotics and disinfectants [11,13]. Consequently, AMR bacterial pathogens may harbour the potential for tolerance to disinfectants, thus influencing the efficacy of C and D practices. The application of disinfectants throughout human and animal sectors, and in environmental facilities such as pollution management facilities, makes this a One Health concern.

Disinfectants target pathogens in a variety of ways depending on their chemical composition. Disinfectants can be grouped into classes, such as alcohols, aldehydes, halogen-releasing, peroxygens, phenolics and quaternary ammonium compound (QAC)-based [14]. The mode of action can vary from targeting the bacterial cytoplasmic membrane [15], inhibiting fatty acid synthesis and denaturing proteins (Table 1) [14]. Bacteria may harbour intrinsic resistance to disinfectants or may acquire resistance, such as following selection pressure [16]. Some species may have an impermeable outer surface membrane, preventing diffusion of the disinfectant into the cytoplasmic space [17]. Should the disinfectant permeate the outer membrane, efflux pumps may actively export the disinfectant out of the cell, thus reducing the concentration within the cell [14]. Properties of the cell surface also influence disinfectant tolerance, such as hydrophobicity and fatty acid composition [18].

**Table 1.** Commonly used disinfectant classes, example chemical composition and potential target sites or modes of action

Disinfectant class	Example chemical composition	Mode of action/target site
Aldehyde	Formaldehyde, glutaraldehyde	Cell wall, protein denaturation and outer membrane (cross-linking)
Halogen-releasing	Iodine, chloride	Cytoplasmic membrane and oxidation of proteins, lipids and nucleic acids
Peroxygen	Peroxymonosulphate, hydrogen peroxide	Cytoplasmic membrane, oxidation of proteins, lipids and nucleic acids and ribosome interference
Phenolic	Chlorocresol, phenolic chlorinated	Cytoplasmic membrane, outer membrane and disruption of enzyme function
QAC	ADBAC, DDAC, BAC	Cytoplasmic membrane and outer membrane

ADBAC, alkyldimethylbenzylammonium chloride; BAC, benzalkonium chloride; DDAC, didecyldimethylammonium dhloride.

Furthermore, bacteria may shield themselves from disinfection via the formation of biofilm. Biofilms are when bacteria encase themselves in a self-produced extracellular polysaccharide and protein matrix (known as EPS) and may persist as aggregates within suspension or adhere to surfaces [19]. The EPS provides a diffusion barrier, whilst metabolic changes within the biofilm community protect the bacteria, such as by reducing growth rate and increasing horizontal gene transfer of genetic material, including resistance determinants [19]. Thus, bacteria in biofilm have been found to show greater tolerance to disinfection than planktonic counterparts [20,21].

The mechanisms utilized by bacteria to evade disinfection vary depending on bacterial species. For example, Gram-positive bacteria typically show greater susceptibility to disinfection than Gram-negative bacteria; the addition of an outer membrane in Gram-negative bacteria aids the cell in tolerating disinfection [17]. Furthermore, evidence shows the use of efflux pumps to be heavily involved in disinfectant tolerance by numerous bacterial species, such as *Staphylococcus aureus* [22,23], *Escherichia coli* [24,25], *Pseudomonas aeruginosa* [26,27] and *Acinetobacter baumannii* [28,29].

Within the UK, disinfectants must be approved against the legislative Department for Environment, Food and Rural Affairs (Defra) disinfectant approvals scheme for use against notifiable disease in livestock. The Defra General Orders (GO) concentration is recommended for general on-farm usage. The method assesses disinfectant efficacy when under ‘dirty’ conditions and when challenged at lower temperatures; both environmental conditions are known to reduce disinfectant efficacy [30,31]. The GO method utilizes a field strain of *Salmonella enterica* subspecies *enterica* serovar Enteritidis as the test organism. Other standardized disinfectant efficacy test methods, such as European Standard EN 1656 (*Quantitative suspension test for the evaluation of bactericidal activity of chemical disinfectants and antiseptics used in the veterinary area*), utilize a range of other bacterial species (*S. aureus*, *Enterococcus hirae*, *Proteus vulgaris* and *Pseudomonas aeruginosa*). Regardless of this bacterial diversity, there is limited knowledge from both these standardized tests and from research studies surrounding disinfectant efficacy against a range of bacterial species, especially those harbouring AMR. Specifically, there is limited knowledge on disinfectant efficacy against organisms of veterinary and public health importance, such as fluoroquinolone-resistant (FQR) *Campylobacter jejuni*, livestock-associated methicillin-resistant *S. aureus* (LA-MRSA), multi-drug resistant (MDR) *E. coli* and vancomycin-resistant *E. faecium* (VRE).

The study aimed to determine the efficacy of a panel of disinfectants against AMR and susceptible strains of four bacterial species of veterinary and public health importance. GO-approved commercial disinfectant products, of various classes, were assessed. This study was performed to determine whether there was a difference in disinfectant susceptibility between bacteria of differing AMR profiles.

## Methods

### Isolate selection

Isolates were selected from the Animal and Plant Health Agency (APHA) culture collection archive in consultation with disease experts (Table 2). A single representative isolate for each species was selected based on their antimicrobial resistance profile; *E. coli* with resistance to HP-CIAs, VRE, LA-MRSA and FQR *C. jejuni*. A single antibiotic-sensitive isolate was also selected for each species. This preliminary investigation was limited to a single isolate for each AMR profile and bacterial species.

**Table 2.** Lists the eight bacterial isolates selected for screening from the APHA culture collection archive

Bacterial species	Isolate	Source	Antimicrobial resistance profile	Isolation year
*FQ-resistant C. jejuni*	S16/0706 – 11 – FZ2210-2182	Broiler	CIP, NAL*	2011
*FQ-sensitive C. jejuni*	NE0900 – 13 – FZ2220-0900	Turkey	Sensitive*	2013
*E. coli* resistant	MSG18-C11	Pig	CIP, NAL‡	2014
*E. coli* sensitive	NCTC 10418	Reference strain	Sensitive	1965
LA-MRSA spa type t011	APHA01	Turkey	β-Lactams, TET‡	2014
MSSA	322/SC290	Unknown	PCN	2008
*E. faecium* (VRE)	C421	Milk	AMP, CHL, ERY, Q-D, TET, VAN†	2003
*E. faecium* (VSE)	C01666	Milk	Susceptible†	2005

β-Lactams, e.g. ampicillin (AMP); chloramphenicol (CHL); quinolones, e.g. nalidixic acid (NAL), including fluoroquinolones, e.g. ciprofloxacin (CIP); tetracyclines, e.g. tetracycline (TET); macrolides, e.g. erythromycin (ERY); streptogramins, e.g. quinupristin-dalfopristin (Q-D); vancomycin (VAN), penicillin (PCN).

*MIC determination was performed as specified by the harmonized monitoring scheme for the EU with interpretation according to 2013 EUCAST epidemiological cut-off values (ECOFF; www.eucast.org) for *Campylobacter* spp.

†MICs were performed using the Sensititre™ EU Surveillance Enterococcus EUVENC AST plate with interpretation according to EUCAST epidemiological cut-off values (ECOFF; www.eucast.org) for *E. faecium*.

‡Based on the presence of resistance genes from whole-genome sequencing data (harboured known genes or mutations confirming phenotypic resistance).

Bacterial characterization was performed prior to this study, via phenotypic testing (i.e. selective agars for selection of *C. jejuni* and *E. coli*) and use of MALDI-TOF (for *Staphylococcus an*d *Enterococcus*). Antimicrobial susceptibility was assessed via broth microdilution assays for *Campylobacter* and *Enterococcus* strains. Whole-genome sequencing data from previous analyses characterized the resistant LA-MRSA isolate ([32], sample accession SAMEA4021843) and resistant *E. coli* MSG18-C11 ([33], sample accession SAMEA4644923).

### Disinfectant selection and preparation

A disinfectant panel of eight commercial disinfectant products, belonging to six disinfectant chemical classes, was selected to represent the common disinfectants in use on farms in the UK; see Table 3. Disinfectants were prepared at the GO-approved dilution rate at the time of testing (Table 3). The diluent used was WHO hard water (calcium chloride, AnalytiChem Belgium NV., Cat. No. CL00.0317; magnesium chloride, VWR, Cat. No. 25108.260), and the disinfectants were prepared and used within 2 h.

**Table 3.** The selected disinfectants, their class and concentration

	Disinfectant ID	Disinfectant class	Defra GO dilution
**1**	Phenolic 1	Phenolic compounds (phenolic chlorinated)	1:50
**2**	Peroxygen 1	Peroxygens (hydrogen peroxide, peracetic acid)	1:180
**3**	QAC 1	QAC including ADBAC and DDAC	1:10
**4**	Aldehyde/QAC 1	Aldehydes/QAC including ADBAC and DDAC	1:25
**5**	Aldehyde 1	Aldehydes	1:40
**6**	Peroxygen 2	Peroxygens (peroxomonosulphate)	1:80
**7**	Iodophor 1	Iodine compounds (iodophor)	1:49
**8**	Chloride 1	Chlorine compounds [sodium dichloroisocyanurate (NaDCC)]	1:360

Dilution rates are shown as 1 part disinfectant to x parts of WHO hard water.

ABDAC, alkyldimethylbenzylammonium chloride; DDAC, didecyldimethylammonium chloride.

### Modified Defra GO disinfectant efficacy method

The GO method follows the British Standard BS6734 : 2004 with slight modifications (based on unpublished work) to accommodate the different growth conditions required for each bacterial species, as the original method tests *Salmonella* Enteritidis PT4 (strain NCTC13665) [34].

The disinfectant test was carried out at a temperature of 4±0.5 °C with a contact time of 30 min and in a 5% w/v yeast suspension. The yeast suspension was made prior to testing (Fresh baker’s Yeast (Pinnacle compressed baker’s yeast made by Mauri Products Ltd.) and WHO hard water).

All isolates were tested against the eight disinfectants, with three biological and five technical replicates per experimental condition.

### Bacterial recovery, culture and incubation conditions

Isolates were stored at −80 °C on Microbank™ vials with cryopreservative (Pro-Lab Diagnostics, Wirral, UK) and were sub-cultured onto 5% sheep blood agar plates (Oxoid Limited, Altrincham, UK) (*E. coli*, *C. jejuni* and *S. aureus*) or tryptone soya agar (TSA) (Oxoid Tryptone Soya Agar, Thermo Fisher Scientific, Cat. No. PO0163A) (*E. faecium*) and incubated at 37±1 °C under either aerobic incubation for 24±3 h for *E. coli*, LA-MRSA and *E. faecium* or micro-aerophilic incubation for 36 to 48 h for *C. jejuni*.

For preparation of McFarland solutions and for enumeration of *E. coli* and *S. aureus,* cultures were diluted in ¼ strength Ringer’s solution (Oxoid™ Ringers Solution Tablets, Thermo Fisher Scientific, Cat. No. BR0052G), *C. jejuni* in peptone-buffered solution (Thermo Scientific™ 0.85% Saline Solution, Cat. No. 13225649) and *E. faecium* in tryptone sodium chloride solution [Oxoid Tryptone (pancreatic digest of casein), Cat. No. LP0042].

Test cultures were grown in nutrient broth no. 2 (Oxoid Nutrient Broth No.2 (Dehydrated), Thermo Fisher Scientific, Cat. No. CM0067b) for *E. coli*, *E. faecium* and *S. aureus*. The test cultures were also plated into blood agar base (BAB) (Oxoid Blood Agar Base (Dehydrated), Thermo Fisher Scientific, Cat. No. CM0055b) (*E. coli* and *S. aureus*) and TSA (*E. faecium*) on the day of the test. *C. jejuni* test cultures were only inoculated into Preston resuscitation broth (Oxoid™ Preston Campylobacter Selective Supplement. Thermo Fisher Scientific, Cat. No. SR0117E) on the day of test and then following incubation (see the ‘Test procedure’ section) were inoculated onto modified charcoal cefoperazone deoxycholate agar (mCCDA) (Oxoid™ CCDA Selective Supplement, Thermo Fisher Scientific, Cat. No. SR0155H) before further incubation.

The *E. coli*, *E. faecium* and *S. aureus* were incubated aerobically at 37±1 °C for 48±3 h in incubation test tubes and plates. *C. jejuni* test tubes were incubated at 37±1 °C for 48±3 h, before inoculation onto mCCDA, followed by a further 24-h incubation.

### Test inoculum preparation

A minimum starting inoculum of >1×10^8^ c.f.u. ml^−1^ was required to be able to confirm the successful efficacy of a disinfectant (i.e. >5 log reduction) following in-test dilution steps. A suitable McFarland solution was prepared for each species to ensure a minimum starting inoculum of >1×10^8^ c.f.u. ml^−1^. McFarland solutions of 5.5 for *C. jejuni*, *E. faecium* and *S. aureus* and 5.0 for *E. coli* were identified as suitable for use in further testing following preliminary screening.

On the day of the test, the McFarland solution was prepared by inoculating the recovered cultures into the relevant diluent (see the ‘Bacterial recovery, culture and incubation conditions’ section) until the required turbidity was obtained, using a densitometer. For plate counts, the McFarland was then diluted to 10^−8^, before 1 ml pour plates were performed in duplicate for the 10^−6^, 10^−7^ and 10^−8^. The plates were incubated alongside the test universals and plates as appropriate (see the ‘Bacterial recovery, culture and incubation conditions’ section). The McFarland solution was used to make the test suspension by inoculating a 5% w/v yeast suspension at a 1 : 24 ratio. The 10^−5^ was used in the disinfectant neutralization validation (DNV) and neutralizer toxicity validation (NTV) validations, as below.

### Disinfectant neutralization and neutralizer toxicity validations

Nutrient broth no.2+5% horse serum [Oxoid Nutrient Broth No.2 (Dehydrated), Thermo Fisher Scientific, Cat. No. CM0067b; Gibco™ Horse Serum, heat-inactivated, New Zealand origin, Fisher Scientific, Cat. No. 10368902] was used as a neutralizer for all disinfectant products except those containing QACs (i.e. QAC 1 and aldehyde/QAC 1). Lecithin (Tween 80, SLS, Cat. No. CHE3854; Saponin, Sigma, Cat. No. 47036–250 f; Lecithin, VWR, Cat. No. 24966.180) was required for successful neutralization of QAC-based disinfectants (i.e. QAC 1 and aldehyde/QAC 1).

NTV was performed to confirm the lack of toxicity of both neutralizers to any of the organisms. In brief, 100 µl of ~1×10^5^ c.f.u. ml^−1^ of relevant organism was aliquoted into each 10 ml neutralizer and 10 ml ¼ strength Ringer’s solution (control), held at 20±2 °C for 5 min before 1 ml aliquots were made into BAB agar pour plates. Counts were performed after incubation for 48 h at 37±1 °C. Neutralizer was deemed non-toxic if counts were greater than half the counts observed on the control plates.

DNV was performed to confirm that the selected test concentrations for all eight disinfectants could be successfully neutralized by the chosen neutralizers after a 30-min contact time. In brief, 5% w/v yeast solution was added 1 : 1 with the highest concentration of disinfectant and held at 4±0.5 °C for 30 min. Then, a 100 µl aliquot was made into a 10 ml neutralizer and held at 20±2 °C for 5 min before 100 µl of ~1×10^5^ c.f.u. ml^−1^ of relevant organism was aliquoted into the neutralizer and 10 ml ¼ strength Ringer’s solution (control). These were held at 20±2 °C for 30 min before 1 ml aliquots were made into BAB agar pour plates. Counts were performed after incubation for 48 h at 37±1 °C. Neutralizer was deemed effective at neutralizing the disinfectant if counts were greater than half the counts observed on the control plates.

### Phenol range finding

The GO method utilizes ultrapure phenol [redistilled phenol Invitrogen UltraPure™ Phenol Cat. No. 15509-037 (Fisher Scientific)] as an internal test control. Three concentrations of phenol are used to exemplify a pass, pass/fail and fail outcome (see the ‘Interpreting results’ section). The concentrations used are specific to the test organism – *Salmonella* Enteritidis in the original GO method – thus, for this investigation, range-finding was required to identify suitable concentrations of phenol for the four species (Table S1, available in the online Supplementary material). A range of concentrations was prepared and tested as per the ‘Test procedure’ section. Suitable fail, pass/fail and pass concentrations for each organism were identified from the range-finding concentrations (Table 4).

**Table 4.** Suitable fail, fail/pass and pass phenol concentrations for each organism

Organism	Phenol concentration		
	Fail	Fail/Pass	Pass
*E. coli* (antibiotic sensitive and resistant)	2.6%	3.0%	3.4%
*E. faecium* (antibiotic sensitive and resistant)	3.4%	4.2%	5.0%
*C. jejuni* (antibiotic sensitive and resistant)	1.0%	2.25%	3.0%
*S. aureus* (antibiotic sensitive)	3.4%	3.8%	4.2%
Methicillin-resistant *S. aureus*	3.4%	4.2%	5.0%

### Test procedure

Disinfectant or phenol was combined in a 1 : 1 ratio with the inoculated 5% w/v yeast suspension and held at 4±0.5 °C. After a 30-min contact time, 100 µl of the test suspension was added to 10 ml of suitable neutralizer, before being placed at 20±2 °C for at least 5 min. Following neutralization, 1 ml aliquots of the neutralized solution were made in five tubes of the relevant resuscitation broth media (see the ‘Bacterial recovery, culture and incubation conditions’ section). For *E. coli*, *E. faecium* and *S. aureus,* a further two 1 ml aliquots were made into pour plates. All tubes and plates (if relevant) were incubated as described in the ‘Bacterial recovery, culture and incubation conditions’ section. For *C. jejuni*, following the incubation of the five tubes, 10 µl from each Preston broth culture was spread plated onto mCCDA agar plates. The plates were then incubated anaerobically for a further 24 h at 37±1 °C.

### Interpreting results

Counts were performed on the 10^−6^, 10^−7^ and 10^−8^ plates to confirm the starting inoculum for all organisms. For *E. coli*, *E. faecium* and *S. aureus*, plate counts were performed to establish c.f.u. ml^−1^ for the disinfectant/phenol test plates, and tubes were observed for turbidity (turbid=growth). For *C. jejuni*, the mCCDA plates were observed for the presence/absence of growth. Tube turbidity for *E. coli*, *E. faecium*, *S. aureus* and *C. jejuni* mCCDA plate results were recorded as growth out of five. A disinfectant was deemed to be effective (i.e. passed, 5 log reduction) if growth (turbidity) in 0/5 or 1/5 tubes was recorded.

### Statistical analysis and graphical presentation

Graphical outputs were generated using RStudio™ (version 2023.09.1) [35] using ggplot2 [36] and stringr [37]. Statistical analyses were performed in RStudio™ (version 2023.09.1) [35], using lattice [38]. Statistical analysis was performed on biological replicate tube result data, i.e. growth in 0, 1, 2, 3, 4 or 5 tubes. Normality was assessed using Shapiro–Wilk (95% confidence interval, *P*≤0.05), which confirmed non-normal distributions. Mann–Whitney *U* test was used to assess significance at a 95% confidence interval (*P*≤0.05). Significance between isolates was assessed either against all disinfectants combined (*n*=48) or against each disinfectant individually (*n*=6 other than peroxygen, *n*=12).

## Results

### Modified Defra disinfectant GO disinfectant efficacy method

#### Overview

Neither neutralizer was toxic towards any of the chosen isolates. All eight disinfectants were successfully neutralized after a 30-min contact time.

**Fig. 1.**
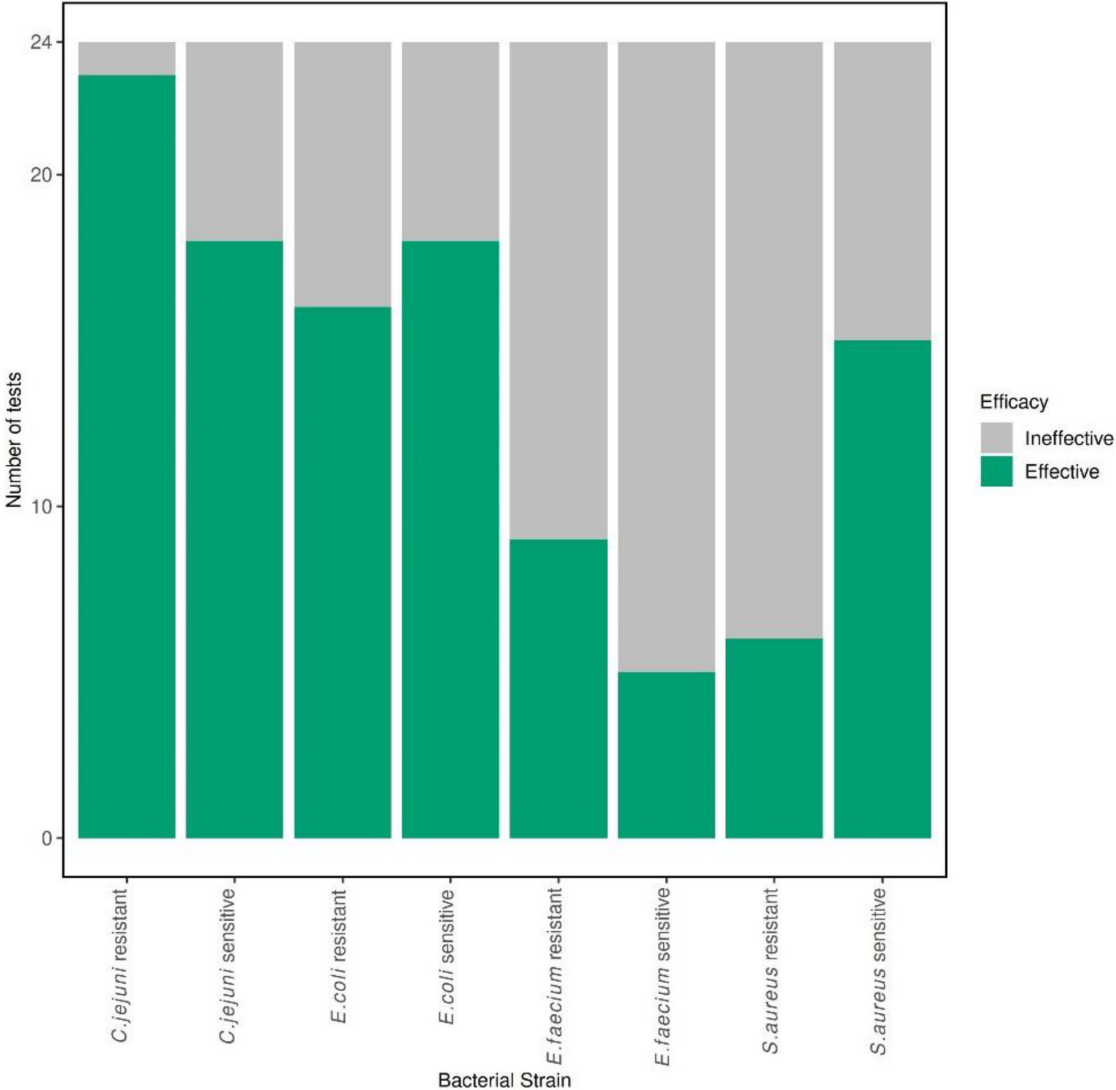
Bar chart depicting the number of pass (effective, green) and fail (ineffective, grey) disinfectant efficacy test results for the sensitive and resistant strains of *E. coli*, *E. faecium*, *S. aureus* and *C. jejuni* individually. Total tests=24.

**Fig. 2.**
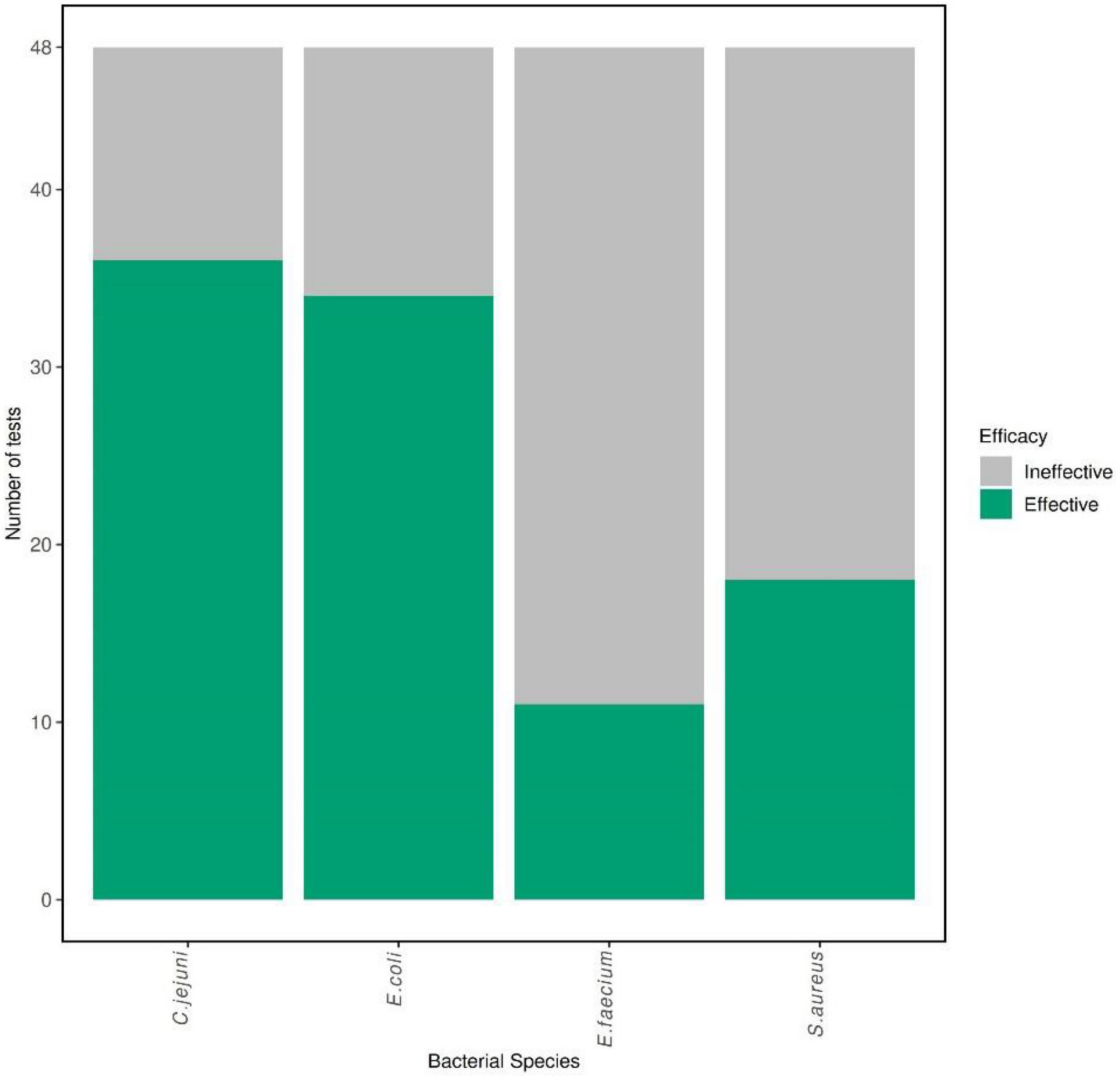
Bar chart depicting the number of pass (effective, green) and fail (ineffective, grey) disinfectant efficacy test results for *E. coli*, *E. faecium*, *S. aureus* and *C. jejuni*, combining sensitive and resistant strains. Total tests=48.

All tests achieved the minimum starting inoculum of >1×10^8^ c.f.u. ml^−1^, with a range between 5.4×10^8^ c.f.u. ml^−1^ and 8.5×10^9^ c.f.u. ml^−1^. Overall, the most susceptible species [i.e. the species with the greatest number of passes/effective disinfection (5-log reduction, <2 turbid tubes)] against all eight disinfectants, following three biological replicates (sensitive and resistant combined, *n*=48), was *C. jejuni* (41/48), followed by *E. coli* (34/48) and *S. aureus* (21/48), with *E. faecium* the least susceptible (14/48) (Fig. 1) . The most susceptible strain against all eight disinfectants, following three biological replicates (*n*=24), was the *C. jejuni* resistant isolate (23/24) (Fig. 2) . The *E. faecium* resistant strain was the least susceptible (5/24) (Fig. 2).

#### Fluoroquinolone-resistant *C. jejuni*

No significant difference (*P*≥0.05, *n*=48) was observed between the susceptibility of the sensitive and resistant strains against the panel of eight disinfectants. Across all eight disinfectant products, in biological triplicate (*n*=24), the *Campylobacter*-sensitive strain passed 18/24 times, whereas the resistant strain passed 23/24 times (Fig. 2), resulting in the resistant strain trending towards being more susceptible.

**Fig. 3.**
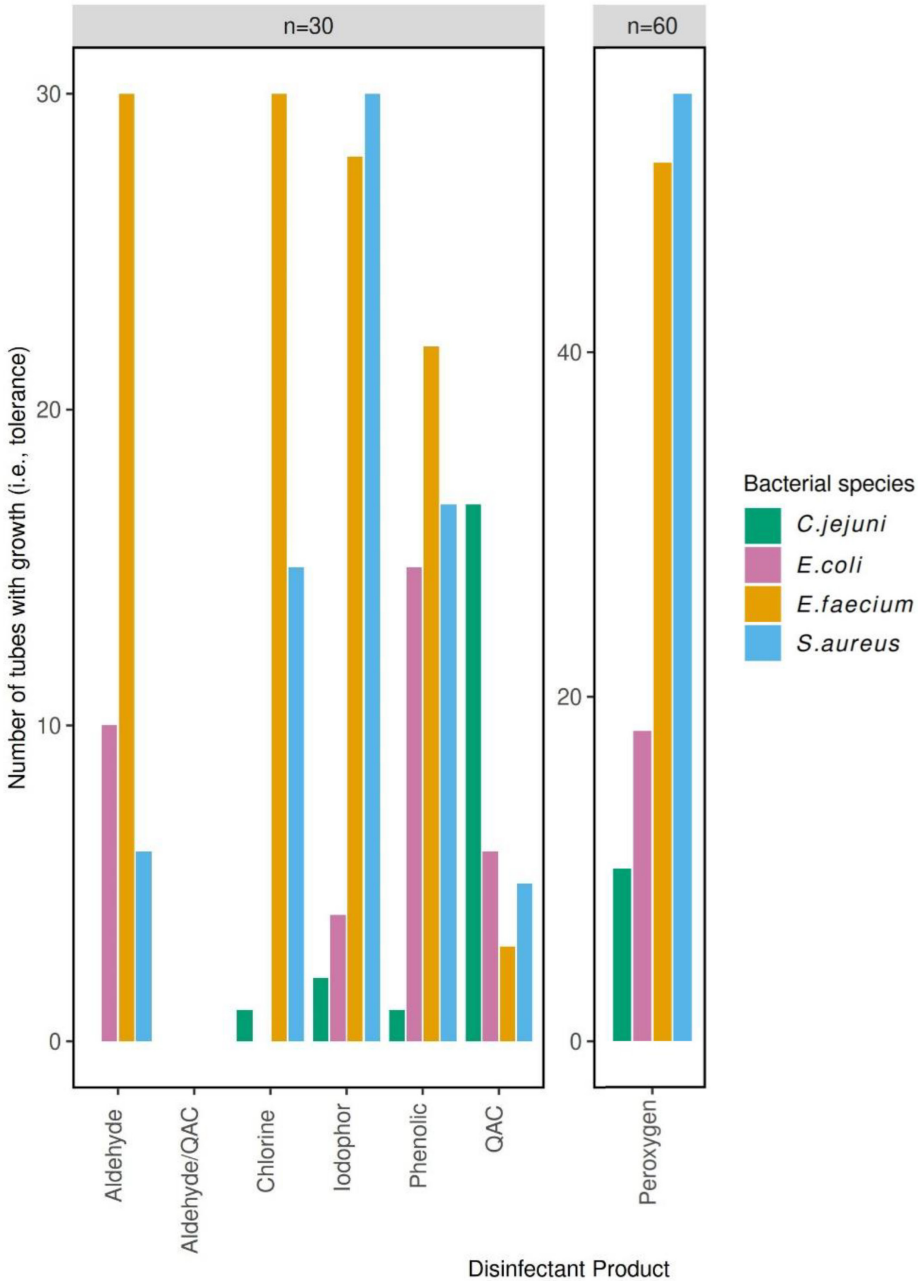
Bar chart depicting the number of tubes with growth (i.e. tolerance) for each disinfectant class for the combined resistant and sensitive *E. coli*, *C. jejuni*, *E. faecium* and *S. aureus* strains. Green, *C. jejuni*; pink, *E. coli*; orange, *E. faecium*; blue, *S. aureus*. Total number of tubes per class of disinfectant (tested in biological triplicate): phenolic, *n*=30; peroxygen, *n*=60; QAC, *n*=30; aldehyde/QAC, *n*=30; aldehyde, *n*=30; iodophor, *n*=30; chlorine, *n*=30.

The least effective disinfectant against the *C. jejuni*-sensitive and -resistant strains combined was the QAC disinfectant (growth in 17/30 tubes) (Fig. 3). The most effective disinfectants against both strains were the aldehyde and aldehyde/QAC disinfectants, with growth in no tubes for both resistant and sensitive strains (Fig. 3).

**Fig. 4.**
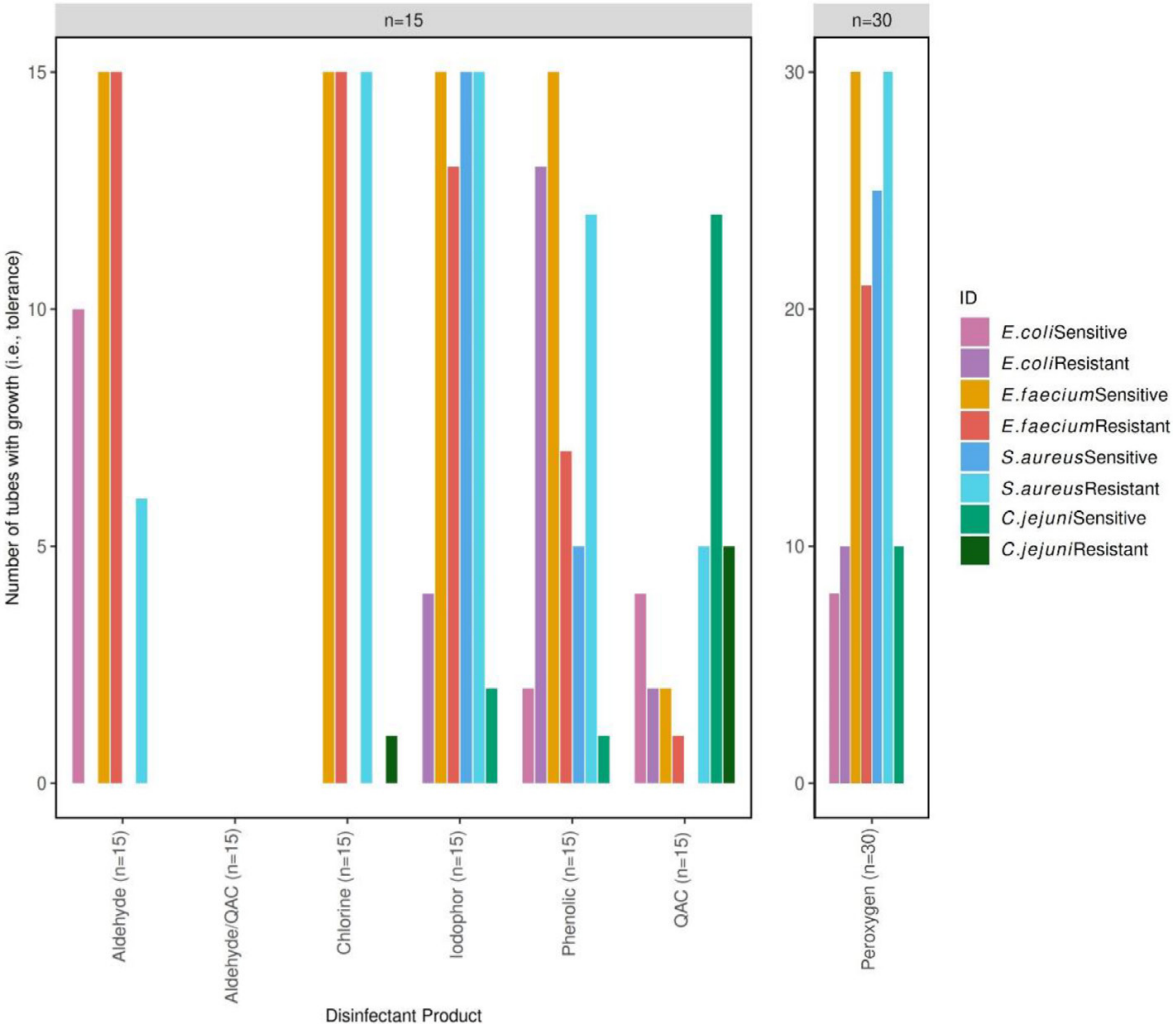
Bar chart depicting the number of tubes with growth (i.e. tolerance) for each disinfectant class for the sensitive and resistant *E. coli* (pink and purple), *C. jejuni* (light green and dark green), *E. faecium* (orange and red) and *S. aureus* (blue and turquoise) strains, respectively. Total number of tubes per class of disinfectant (tested in triplicate): phenolic, *n*=15; peroxygen, *n*=30; QAC, *n*=15; aldehyde/QAC, *n*=15; aldehyde, *n*=15; iodophor, *n*=15; chlorine, *n*=15.

However, no significant difference [*P*≥0.05, *n*=6 (peroxygen *n*=12)] was observed between the *C. jejuni*-sensitive and -resistant strains for any of the disinfectants. Whilst not significant, the sensitive strain was less susceptible than the resistant strain for the QAC, phenolic, peroxygen and iodophor disinfectants, with growth in tubes decreasing from 12/15 to 5/15, 1/15 to 0/15, 10/30 to 0/30 and 2/15 to 0/15, respectively (Fig. 4). On the other hand, the *Campylobacter*-resistant strain was less susceptible to the chlorine disinfectant than the sensitive (1/15 and 0/15 growth in tubes, respectively (Fig. 2).

#### Multi-drug resistant *E. coli*

No significant difference (*P*≥0.05, *n*=48) was observed between the susceptibility of the sensitive and resistant *E. coli* strains across the panel of disinfectants. For all eight disinfectant products, the *E. coli* sensitive strain was marginally more susceptible than the resistant strain, producing pass results 18/24 times (Fig. 2) vs. 16/24 times for the resistant strain (Fig. 2).

The least effective disinfectant against the *E. coli*-sensitive and -resistant strains combined was the phenolic disinfectant (growth in 15/30 tubes) (Fig. 3). The most effective disinfectants against both strains were the chloride and aldehyde/QAC disinfectants, with both the resistant and sensitive strains showing 0/15 growth in tubes (Fig. 3).

No significant difference [*P*≥0.05, *n*=6 (peroxygen *n*=12)] in susceptibility was observed between the *E. coli*-sensitive and -resistant strains for any of the disinfectants. Whilst not significant, the sensitive strain was found to be less susceptible to the aldehyde product than the resistant strain, with growth in tubes decreasing from 10/15 (sensitive) to 0/15 (resistant) (Fig. 4). However, the *E. coli* resistant strain was less susceptible to the phenolic and iodophor disinfectants, with growth in tubes increasing from 2/15 (sensitive) to 13/15 (resistant) and 0/15 to 4/15, respectively (Fig. 4).

#### Livestock-associated methicillin-resistant *S. aureus*

There was a significant difference (*P*<0.05, *n*=48) between the susceptibility of the sensitive and resistant *S. aureus* strains across the panel of disinfectants. Across all eight disinfectant concentrations, the *Staphylococcus*-sensitive strain passed 15/24 times (Fig. 2). The resistant strain passed 6/24 times (Fig. 2); therefore, the sensitive strain was more susceptible to the disinfectants.

The least effective disinfectant against the *S. aureus*-sensitive and -resistant strains combined was the iodophor disinfectant (growth in all 30 tubes) (Fig. 3). The most effective disinfectant against both strains was the aldehyde/QAC disinfectant (no growth in all 30 tubes) (Fig. 3).

Statistical significance between the susceptibility of the sensitive and resistant strains could not be calculated for the chloride disinfectant due to each strain conferring perfect efficacy or failure (15/15 tubes for the resistant strain compared to 0/15 for the sensitive strain) (Fig. 4). No significant difference (*P*≥0.05) in susceptibility was observed between the *S. aureus*-sensitive and -resistant strains for any of the other disinfectants. The number of tubes with growth was equal for both strains for the iodophor (15/15) and aldehyde/QAC (0/0) disinfectants (Fig. 4). Whilst not significant, the resistant strain was less susceptible than the sensitive strain for the phenolic (12/15 vs. 5/15), peroxygen (30/30 vs. 25/30), QAC (5/15 vs. 0/15), and aldehyde (6/15 vs. 0/15) (Fig. 4).

#### Vancomycin-resistant *E. faecium*

There was no significant difference (*P*≥0.05, *n*=48) between the susceptibility of the sensitive and resistant *E. faecium* strains amongst the panel of disinfectants. Across all eight disinfectant concentrations, the *Enterococcus*-sensitive strain passed 5/24 times (Fig. 2). The resistant strain passed 9/24 times (Fig. 2); therefore, the resistant strain was more susceptible to the disinfectants.

The least effective disinfectants against both *E. faecium* strains were the chlorine and aldehyde products, with growth in all 30 tubes (Fig. 3). The *E. faecium* was most susceptible towards the aldehyde/QAC disinfectant, with growth observed in none of the tubes for either resistant or sensitive strain (Fig. 3).

No significant difference (*P*≥0.05) in susceptibility was observed between the *E. faecium*-sensitive and -resistant strains for any of the disinfectants. The number of tubes with growth was equal for both the sensitive and resistant isolates for the chloride (15/15), aldehyde/QAC (0/15) and aldehyde (15/15) disinfectants (Fig. 4). Whilst not significant, the sensitive strain was less susceptible than the resistant strain for the phenolic (15/15 vs. 7/15), peroxygen (30/30 vs. 21/30), QAC (2/15 vs. 1/15) and iodophor (15/15 vs. 13/15) disinfectants (Fig. 4).

## Discussion

The presence of AMR pathogens is a One Health concern. The potential for co- and cross-resistance between antibiotic resistance and disinfectant tolerance should be considered to ensure appropriate implementation of C and D protocols. The efficacy of C and D protocols is already known to vary depending on many environmental factors such as surface, the presence of organic matter, temperature, contact time, disinfectant concentration and species of target organism [30]. However, a greater understanding is required to understand the role of AMR mechanisms on disinfectant tolerance.

The current study aimed to compare and evaluate the tolerance of FQR *C. jejuni*, LA-MRSA, MDR *E. coli* and VRE with their antibiotic-sensitive counterparts, in the presence of organic matter, to eight commercially available disinfectants, covering seven different chemical classes. A potential role of AMR on disinfectant tolerance was only observed between the resistant and sensitive *S. aureus* when comparing the susceptibility of the two strains against all eight disinfectant products collectively. However, no role could be ascertained for individual disinfectant classes. Additionally, no role of AMR on disinfectant tolerance could be determined for *C. jejuni*, *E. coli* or *E. faecium*, across the eight products collectively nor individually.

Whilst a role of AMR on disinfectant susceptibility was identified for *S. aureus* overall, the role of disinfectant class was unclear. This is possibly due to limitations of the study, such as the inclusion of only two genetically distinct strains and a small panel of disinfectant products. Despite this, a role of AMR on *S. aureus* disinfectant susceptibility has been found in other studies [8,39, 40]. Sarwar *et al*. [40] [38] found that MRSA isolates that harbour qac genes require higher concentrations of select disinfectants to be effective. They suggest the *qac* gene family, which encodes multidrug efflux pumps, plays a role in exporting toxic molecules out of the bacterial cell [41]. Likewise, Mycock *et al*. [39] identified plasmid-mediated tolerance of MRSA to povidone-iodine. However, other studies found AMR *S. aureus* to be no more susceptible to phenolic products than sensitive strains [42]. Inclusion of a larger isolate panel and a more diverse range of disinfectant products, with the addition of genetic analysis, would improve conclusions on the role of AMR in *S. aureus* disinfectant susceptibility.

As found here, multiple studies found no difference between the disinfectant susceptibility of antibiotic-sensitive and -resistant *E. faecium* strains [43,45]. However, a study by Schwaiger *et al*. [46] observed greater tolerance in antibiotic-resistant *E. faecium* strains, particularly to didecyldimethylammonium chloride (a commonly used component of QAC disinfectants). However, their methodology differed vastly from this study, utilizing a standardized broth microdilution assay with a disinfectant contact time of up to 72 h, without the inclusion of organic matter. The disinfectant concentration range also differed, making comparisons difficult to draw. As previously described, organic matter causes a reduction in disinfectant efficacy; therefore, the lack of organic matter in their study reduces the ability to draw comparisons between the two studies but also limits the application of their results to ‘real-world’ scenarios. Furthermore, the use of a longer contact time also limits comparison with the current study and may also influence the interpretation of results due to a longer period of recovery for the bacteria following exposure to subinhibitory concentrations of disinfectant. Consequently, careful consideration should be given to the *in vitro* test conditions when trying to ascertain the role of AMR on disinfectant tolerance.

It may be anticipated that disinfectant class influences the role of AMR on disinfectant tolerance. The mode of action of a disinfectant varies depending on its class. For example, aldehydes act by targeting cytoplasmic membrane proteins, causing membrane damage, coagulation of inner cell components and enzyme inhibition [12,47]. However, iodides passively diffuse through the cell membranes, leading to cell death due to oxidation of intracellular components [48]. These modes of action commonly rely on mechanisms that are like those utilized by antibiotics. Consequently, co-resistance between antibiotics and different disinfectant classes has been increasingly observed [6]. However, the current study did not support these findings.

No role of AMR on disinfectant tolerance was identified between the MDR *E. coli* and antibiotic-sensitive *E. coli* strains for any disinfectant product. However, tolerance towards specific classes of disinfectant may have been anticipated. For example, phenolic disinfectants act by initiating cell death following leakage of metabolites through efflux pumps via the cell wall [49]. Therefore, as the MDR *E. coli* strain harboured resistance to nalidixic acid, which plays a role in the downregulation of efflux pumps [50], greater tolerance than the sensitive strain may have been observed. However, further investigation would be required to confirm this.

Likewise, cross-resistance has been identified between QAC disinfectants and fluoroquinolones [51,55], due to the influence of multidrug efflux pumps. However, the *C. jejuni* FQR strain used in this study did not show higher tolerance towards QACs. Yet, no difference in susceptibility was observed. These previous studies identified the potential relationship between QACs and fluoroquinolones in a variety of other bacterial species. Therefore, a relationship between QACs and fluoroquinolones may not play the same role in *Campylobacter*. Beier *et al*. [56] found no relationship between antimicrobials and disinfectants in FQR *C. jejuni*. Furthermore, they found high susceptibility to QACs, opposing what was observed in this study. However, again, they examined disinfectant susceptibility in standardized broth microdilution assays for 42 h without the inclusion of organic matter. The lack of evidence of cross-resistance between antibiotics and disinfectants may relate to the *Campylobacter* serovar. Different serovars and clonal complexes of *C. jejuni* have been documented to exhibit varying growth capabilities and virulence in a range of environments [57,58]. Consequently, the chosen strains could be expected to exhibit varying tolerance towards disinfectants due to serovar, irrespective of antibiotic tolerance.

The disinfectants were tested at the Defra disinfectant approvals GO concentration at the time of the investigation. This concentration is formally approved against the test organism *Salmonella* Enteritidis. However, the concentration of disinfectant required to be effective (>5 log reduction) against different bacterial species is known to vary [59,60]. The results obtained in this study support this statement, as the GO concentration was not always effective against the four bacterial species studied. The most susceptible species was *C. jejuni*, followed by *E. coli*, *S. aureus* and *E. faecium*. The Gram-positive bacteria (*S. aureus* and *E. faecium*) were more tolerant to the disinfectants than the Gram-negative bacteria (*C. jejuni* and *E. coli*); this opposes research that suggests vice versa [17,31]. The addition of the outer cell membrane in Gram-negative bacteria may protect from disinfectants such as QACs [14,17]. However, both Willinghan *et al*. [61] and McDonnell and Russell [8] also found *S. aureus* to be highly resistant to disinfectants.

Furthermore, numerous studies have identified variations in the effectiveness of disinfectants dependent not only on the target organism but also on chemical class [59,60]. Again, the same was identified within this study, with cross-species variation observed. *E. coli* was found to be most tolerant against phenolics yet susceptible to chlorines, whereas *C. jejuni* was found to be most tolerant to QACs but highly susceptible to aldehydes. Likewise, *S. aureus* was also most susceptible to aldehydes and most tolerant to iodophors. Lastly, *E. faecium* was most tolerant to chlorines and most susceptible to QACs. In contrast to Rodgers *et al*. [60], this study found *S. aureus* to be highly tolerant to peroxygen disinfectants. However, Rodgers *et al*. [60] examined disinfectant efficacy at room temperature, as opposed to 4 °C in this study. Higher temperature has previously been found to increase disinfectant efficacy [62,63]. In agreement with previous studies, *E. faecium* was found to be highly susceptible to QACs [59].

The findings of this study highlight the variation in disinfectant susceptibility observed between and within bacterial species due to differing strain features (i.e. serovar, genetic determinants and source of isolation). This highlights the importance of understanding the target organism when utilizing disinfectants in the field. Further study could highlight whether certain chemical classes are more appropriate for use against specific bacterial species than others. Additionally, understanding the role of external factors such as temperature, surface, contact time and the presence of organic matter is imperative to ensure appropriate and effective disinfection occurs. Different conditions should be considered depending on the purpose or application, such as for use on farms vs. in veterinary or clinical settings. Thus, the *in vitro* method in which disinfectants are assessed should be carefully considered.

Only one sensitive and one resistant strain for each species was included in this study. These strains were not isogenic and thus may vary genetically in a variety of ways. Such variation may include serovar, clonal complex or the presence of genetic components involved in a variety of metabolic and virulence functions. To counter some of this variation, isolates were chosen to be as comparable as possible, considering factors such as sequence type, source of isolation and species of source (i.e. poultry, cattle). The selection of the strains from the APHA culture collection may have limited the applicability of the results. Whilst covering a diverse range of sources in the UK agricultural and veterinary fields, these strains may represent a limited diversity from other settings. Further study should be performed to investigate the genomic characteristics of the strains, particularly focusing on disinfectant mechanisms.

## Conclusion

The results obtained in this study suggest a potential relationship between disinfectant tolerance and antibiotic resistance for *S. aureus*, yet not for *E. coli*, *C. jejuni* or *E. faecium*. The role of disinfectant class on tolerance was unclear. Disinfectant efficacy was found to vary for different bacterial species, dependent on disinfectant class. Further research should be performed to examine the genomic characteristics involved in disinfectant tolerance and to understand in greater depth the influence of environmental conditions on disinfectant efficacy.

## Supplementary material

10.1099/acmi.0.001098.v4 Uncited Table S1.

## Abbreviations

AMR: antimicrobial-resistant
BAB: blood agar base
Defra: Department for Environment, Food and Rural Affairs
DNV: disinfectant neutralization validation
EPS: extracellular polysaccharide and protein matrix
EU: European Union
FQR: fluoroquinolone-resistant
GO: General Orders
HP-CIA: high-priority critically important antibiotics
LA-MRSA: livestock-associated methicillin-resistant *Staphylococcus aureus*
mCCDA: modified charcoal cefoperazone deoxycholate agar
MDR: multi-drug resistant
NTV: neutralizer toxicity validation
QAC: quaternary ammonium compound
TSA: tryptone soya agar
VRE: vancomycin-resistant *Enterococcus faecium*
WHO: World Health Organization
