# Supplementary material for: Influence of antibiotic resistance on disinfectant tolerance of Escherichia coli, Staphylococcus aureus, Enterococcus faecium and Campylobacter jejuni
Source: Access Microbiol. 2025 Dec 22;7(12):001098.v4. doi: 10.1099/acmi.0.001098.v4 (PMC12721599; doi:10.1099/acmi.0.001098.v4)
Supplement: Uncited Table S1. [file acmi-7-01098-s001.pdf]

**Range finding of appropriate concentrations of ultrapure phenol for use as an internal control in the General Orders (GO) method against *Enterococcus faecium*, *Campylobacter jejuni*, *E. coli* and LA-MRSA**

A range of concentrations of phenol were identified for each test organism (**Error! Reference source not found.**). These were chosen based on previous internal studies (unpublished) where the same bacterial species (different strains) were tested against phenol. These concentrations were then tested under a modified version of the GO method, as described in the main publication, against the studies strains. Tests were performed in biological triplicate. Adjustments were made to the phenol concentrations as required (i.e. lower concentrations removed when clearly too weak, or higher concentrations added when pass results were not observed).

*Table 1 describes the chosen concentrations of phenol used in range finding experiments, to find a pass, pass/fail and fail concentration for each bacterial species*

| Bacterial Species | Chosen Phenol Concentrations |
|---|---|
| *Escherichia coli* | 2.6% (1.3g), 3% (1.5g), 3.4% (1.7g), 3.8% (1.9g), 4.2% (2.1g) |
| *Campylobacter jejuni* | 1% (0.5g), 2% (1g), 2.25% (1.125g), 2.5% (1.25g), 3% (1.5g) |
| Livestock Associated *Staphylococcus aureus* | 2.6% (1.3g), 3% (1.5g), 3.4% (1.7g), 3.8% (1.9g), 4.2% (2.1g), 4.6% (2.3g) |
| *Enterococcus faecium* | 3.4% (1.7g), 3.8% (1.9g), 4.2% (2.1g), 4.6% (2.3g), 5.0% (2.5g) |

Table 1 shows the tube (or plate for *Campylobacter*) results for each organism. Both *E. coli* isolates produced the same results with fails at 2.6% and 3% and passes at 3.4%, 3.8% and 4.2%. Both *Campylobacter* isolates produced fails at 1.0% and 2.0% and a pass/fail at 2.25% and 2.5% and a pass at 3.0%. The sensitive *Staphylococcus* (MSSA) was highly tolerant at lower concentrations; thus, the two lowest concentrations were dropped after the first replicate and the concentrations increased. The 4.2% and 4.6% passed whilst the remaining lower concentrations all failed. The resistant *Staphylococcus* (MRSA) required slightly higher concentrations to pass - 4.8% and 5.0%. *Enterococcus* failed at 3.4% and 3.8%, pass/failed at 4.2% and passed at 4.6% and 5.0%.

*Table 1 shows the tube (or plate for Campylobacter) results from the biological triplicate phenol range finding for each strain. Results are reported as growth in five tubes/plates. Growth in 2 or more tubes/plates is deemed a fail/ineffective. NT – not tested.*

| **Organism** | **Repeat** | **Phenol Concentration** | | | | | |
|---|---|---|---|---|---|---|---|
| | | **2.8%** | **3.2%** | **3.6%** | **3.8%** | **4.2%** | **-** |
| ***Escherichia coli*** resistant | **1** | **5/5** | **5/5** | **0/5** | **0/5** | **0/5** | **-** |
| | **2** | **5/5** | **5/5** | **0/5** | **0/5** | **NT** | **-** |
| | **3** | **5/5** | **5/5** | **0/5** | **0/5** | **NT** | **-** |
| | | **2.8%** | **3.2%** | **3.6%** | **3.8%** | **4.2%** | **-** |
| ***Escherichia coli*** sensitive | **1** | **5/5** | **5/5** | **0/5** | **0/5** | **0/5** | **-** |
| | **2** | **5/5** | **5/5** | **0/5** | **0/5** | **NT** | **-** |
| | **3** | **5/5** | **5/5** | **0/5** | **0/5** | **NT** | **-** |
| | | **1%** | **2%** | **2.25%** | **2.5%** | **3%** | **-** |
| **Campylobacter *jejuni*** | **1** | **5/5** | **3/5** | **2/5** | **0/5** | **0/5** | **-** |
| | **2** | **5/5** | **4/5** | **4/5** | **4/5** | **0/5** | **-** |

| | | | | | | | |
|---|---|---|---|---|---|---|---|
| resistant | **3** | **5/5** | **4/5** | **2/5** | **0/5** | **0/5** | **-** |
| | | 1% | **2%** | **2.25%** | **2.5%** | **3%** | **-** |
| **Campylobacter *jejuni*** sensitive | **1** | **5/5** | **5/5** | **3/5** | **0/5** | **0/5** | **-** |
| | **2** | **4/5** | **5/5** | **4/5** | **3/5** | **0/5** | **-** |
| | **3** | **5/5** | **5/5** | **1/5** | **0/5** | **0/5** | **-** |
| | | **3.4%** | **3.8%** | **4.2%** | **4.6%** | **4.8%** | **5.0%** |
| ***Staphylococcus aureus*** Methicillin resistant | **1** | **5/5** | **5/5** | **5/5** | **0/5** | **0/5** | **0/5** |
| | **2** | **5/5** | **5/5** | **1/5** | **0/5** | **0/5** | **0/5** |
| | **3** | **5/5** | **5/5** | **3/5** | **2/5** | **0/5** | **0/5** |
| | | **2.6%** | **3%** | **3.4%** | **3.8%** | **4.2%** | **4.6%** |
| ***Staphylococcus aureus*** Methicillin sensitive | **1** | **5/5** | **5/5** | **5/5** | **5/5** | **0/5** | **NT** |
| | **2** | **NT** | **NT** | **5/5** | **2/5** | **0/5** | **0/5** |
| | **3** | **NT** | **NT** | **5/5** | **2/5** | **0/5** | **0/5** |
| | | **3.4%** | **3.8%** | **4.2%** | **4.6%** | **5.0%** | **-** |
| ***Enterococcus faecium*** resistant | **1** | **5/5** | **5/5** | **5/5** | **0/5** | **0/5** | **-** |
| | **2** | **5/5** | **5/5** | **1/5** | **0/5** | **0/5** | **-** |
| | **3** | **5/5** | **5/5** | **1/5** | **0/5** | **0/5** | **-** |
| | | **3.4%** | **3.8%** | **4.2%** | **4.6%** | **5.0%** | **-** |
| ***Enterococcus faecium*** sensitive | **1** | **5/5** | **5/5** | **5/5** | **0/5** | **0/5** | **-** |
| | **2** | **5/5** | **5/5** | **5/5** | **1/5** | **0/5** | **-** |
| | **3** | **5/5** | **5/5** | **1/5** | **0/5** | **0/5** | **-** |

In conclusion, this range finding study was performed to identify suitable phenol concentrations to produce a pass, pass/fail and fail result against antibiotic resistant and susceptible isolates of *Enterococcus faecium*, *Campylobacter jejuni*, *E. coli* and LA-MRSA. Following repeat testing, phenol concentrations of 2.8%, 3.2% and 3.6%, respectively, were chosen for *E. coli*, 1.0%, 1.25% and 3.0% for *Campylobacter*, 3.4%, 3.8% and 4.2% for MSSA, 3.4%, 4.2%, and 5.0% for MRSA, and 3.4%, 4.2% and 5.0% for *Enterococcus.*
